# Malnutrition among the aged population in Africa: A systematic review, meta-analysis, and meta-regression of studies over the past 20 years

**DOI:** 10.1371/journal.pone.0278904

**Published:** 2022-12-09

**Authors:** Temesgen Muche Ewunie, Habtamu Endashaw Hareru, Tadesse Mamo Dejene, Semagn Mekonen Abate

**Affiliations:** 1 Department of Human Nutrition, College of Medicine and Health Science, Dilla University, Dilla, Ethiopia; 2 School of Public Health, College of Medicine and Health Science, Dilla University, Dilla, Ethiopia; 3 Department of Public Health, Asrat Woldeyes Health Science Campus, Debre Birhan University, Debre Birhan, Ethiopia; 4 Department of Anesthesiology, College of Medicine and Health Science, Dilla University, Dilla, Ethiopia; Wollo University, ETHIOPIA

## Abstract

**Background:**

Nowadays, malnutrition among the advanced age (60 years and older) population is becoming a public health problem worldwide, especially in low-income countries including Africa. Hence, the prevalence in Africa is still not known. So, this review aimed to assess the pooled prevalence of under-nutrition among the advanced age population in Africa.

**Methods:**

A study search was carried out using databases (such as African Journals Online, Web of Science, Global Index Medicus, Embess, and PubMed) and gray literature following PRISMA guidelines from April 20, 2022, to May 30, 2022, with no restriction on date of publication. We used a standardized extraction format to compile eligible studies as per the inclusion criteria. Then, systematic review and meta-analysis were employed using a random effect model to obtain the pooled prevalence of malnutrition among aged population living in Africa. The counter-funnel plot and at the 5% significance level, Egger’s test and Begg’s test were used to check for publication bias. Furthermore, a meta-regression analysis was carried out to identify the relationship between the outcome of interest and different predictors.

**Results:**

A total of 731 studies were identified and 28 met the inclusion criteria, which were conducted in 17 African countries. The pooled prevalence of under-nutrition in Africa was 17% (95%CI; 13.5–20.6). The prevalence of malnutrition among the elderly varied significantly across countries, ranging from 1.8% (95% CI; 0.96–2.63) in South Africa to 39.47% (95% CI; 31.70–47.24) in Kenya. According to meta-regression analysis, the likelihood of a malnutrition problem would be reduced by a factor of 9.84 (β = -9.84, 95 percent CI; _-14.97, -4.70, P = 0.00) in upper-middle income countries. In addition, based on the publication year, malnutrition has decreased by a factor of 0.75 (β = -0.75, 95%CI:-1.49, -0.01, P = 0.04) from 1998 to 2021.

**Conclusion:**

There is a high prevalence of malnutrition among the aged population. So, this underserved population should be targeted for intervention programs and/or integrated into maternal and child nutrition programs.

## Introduction

The number of geriatric population aged 60 and over is dramatically rising globally, and by 2050, it is expected to double, from an estimated 1 billion (12%) in 2020 to 2 billion (22%) [1, 2]. Increasing longevity is now creating a new challenge and these segments of the population are more vulnerable to malnutrition [3], because aging may come with cognitive and physical decline, depressive symptoms, and emotional variations [4]. These factors collectively increase the prevalence of malnutrition among elders [5]. Therefore, malnutrition among the geriatric population is a public health problem in low and middle-income countries [6–8] such problem is more serious in developing countries because of poverty, low dietary diversity [9], and comorbidity [10]. Furthermore, evidence suggests that malnutrition is more prevalent in the geriatric population, but little attention has been given [11].

Malnutrition among elders is becoming one of the major public health concerns in developing countries that cause high numbers of mortality [12]. The problem is underestimated and persistent in low-income countries, including the continent of Africa [13].

Despite numerous studies that have been conducted on malnutrition, data on the prevalence of malnutrition among the geriatric population is limited in the African continent that could be used for nutritional intervention on such segment of the population. Therefore, this systematic review and meta-regression aimed to assess the prevalence of malnutrition among the geriatric population in Africa. Hence, evidence generated from this review will be used by nutrition program implementers, policymakers, stakeholders, and health experts to achieve sustainable development goals.

## Methods and materials

### Search strategies and selection process

We followed PRISMA guidelines [14] to search articles using different electronic databases like Web of Science, African journals online, Global Index Medicus, Embess, and PubMed without date restriction from April 20, 2022, to May 30, 2022. The search process included the following key terms like:-”under-nutrition”, “malnutrition”, “nutritional status”, geriatric”, “elders”, “older”, “advanced age population” along with the names of each African nation. In order to not to miss studies, we searched references of relevant articles. Following the title and abstract screening process, studies that met the eligibility criteria underwent full text review, and EndNote X9 software was used to maintain citations and manage duplicate articles in the review process.

### Eligibility criteria

Studies conducted in Africa and reported the prevalence of under-nutrition, used measurements like body mass index (BMI) and a mini-nutritional assessment tool (MNA), observational study, conducted on the aged population (elderly), and published in English were included. But, we excluded studies that did not contain sufficient data on prevalence, conducted in countries other than African nations. In addition, studies with incomplete data and not accessible, published other than English language were not included in the analysis.

### Outcome measurements

Studies that used either BMI and/or MNA measurement to assess malnutrition in an aged population were included. Nonetheless, in the studies that used both MNA and BMI measurements, the MNA measurement result was used for this review. Thus, malnutrition among the geriatric population is the outcome of interest which was dichotomized in to malnourished or not which was assessed by either BMI or MNA. Based on 1),BMI;- Those who had BMI less than 18.5 kg/m were considered as malnourished and those whose BMI was 18.5 Kg/m and above were not malnourished [15], 2),MNA:- the MNA scale was the other assessment methods that consisted of 4 nutritional areas: anthropometric measurement, dietary questionnaire, global assessment and subjective assessment. Then the sum of the score of MNA categorized into malnourished (if the score < 17), and not malnourished (if the score 17 and above) [16].

### Data extraction

Identification of eligible studies were done using Microsoft excel sheet by three independent researchers (TME, TMD, and HEH) and disagreement was resolved in all process by discussion with the fourth researcher (SMA). For each eligible study, the following information was extracted: the name of the author(s), the year of publication, the study country, the outcome measurement (BMI, MNA), the study design, the response rate, the sample size, and the prevalence of malnutrition with a 95% confidence interval. In addition, the countries’ income economy level, based on the recent World Bank economic classification [17], was included in the data extraction process.

### Study quality assessment

The quality of studies was assessed by two reviewers (TME and HEH) rigorously using the Newcastle–Ottawa scale for cross-sectional studies [18]. The quality tool consists of a total of 10 questions assessing different aspects of the study, and each question is scored as yes (1), no (0), or not applicable (N/A). Finally, those articles that scored six or above out of a total of 10 criteria were included in this review.

### Statistical analysis

The outcome was the proportion of malnourished elders and was dichotomized into malnourished and not malnourished. The results were presented as a percentage with a 95% confidence interval (CI), and the analysis was carried out in STATA version 16 software using the metan function in the meta-package. The I^2^ statistic was checked to determine the studies’ heterogeneity, which describes the percentage of total variation among studies that was due to heterogeneity rather than chance. When I^2^ exceeds 75%, homogeneity was considered [19]. A random-effect model was used and subgroup analysis was conducted to manage heterogeneity among studies using measurements, and the country’s economic status. Sensitivity analysis was performed to determine the effect of a single study on the pooled estimate of outcome. Potential publication bias was checked by a funnel plot through observational assessment. In addition, the bias was checked by Egger’s test and Begg’s test at a 5% significance level. Then, a non-parametric trim and fill analysis was performed to manage the publication bias [20]. A meta-regression was performed to measure the dependency of outcome on the predictors and to investigate potential effect modifiers that explain any heterogeneity effect among studies. As an assumption of meta-regression analysis, a minimum of ten studies required to perform meta-regression and to see linear-correlation of outcome variable and selected predictors.

## Results

**Fig 1:**
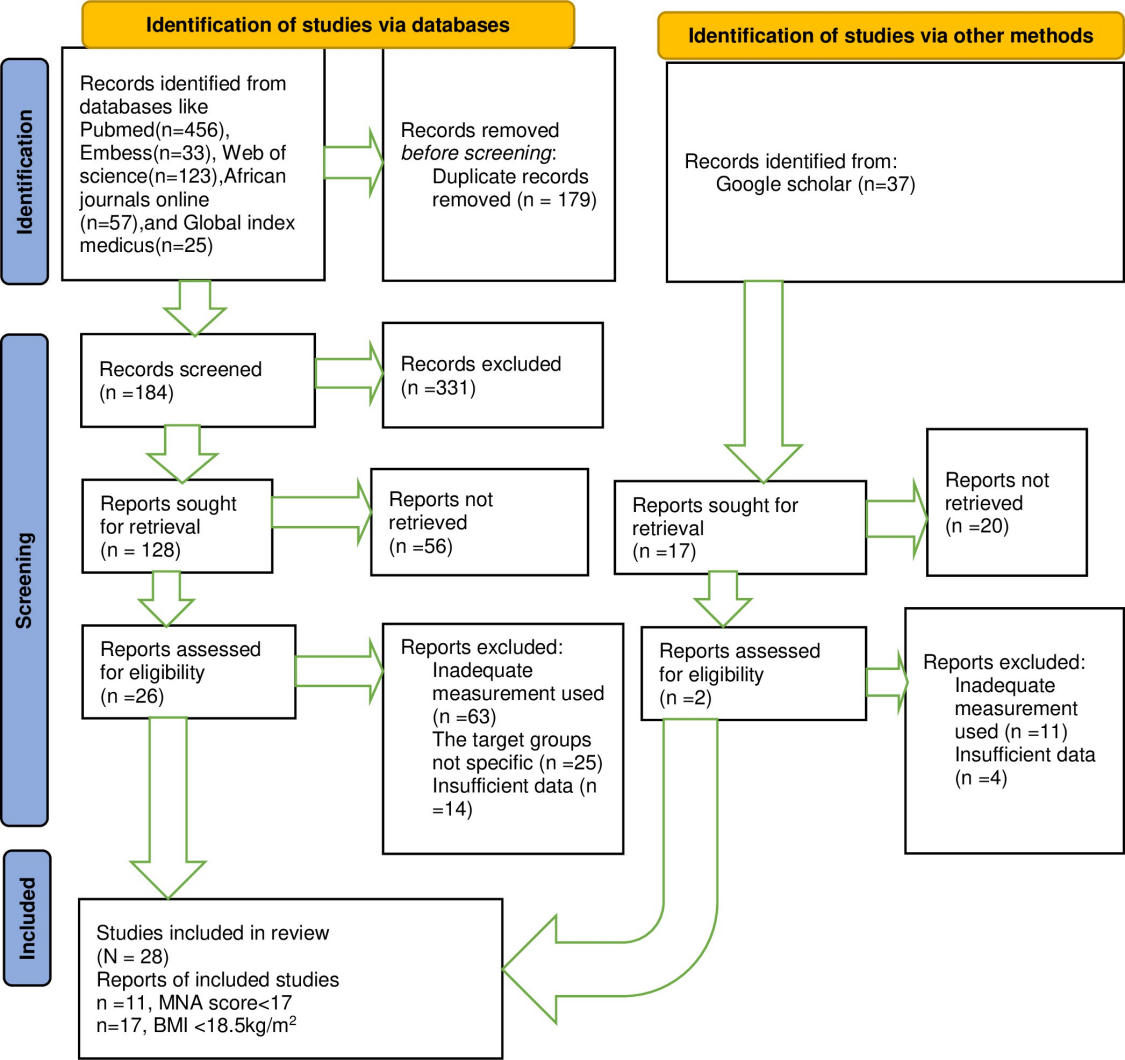
A PRISMA flow diagram for systematic reviews, met analysis and meta-regression of included studies.

A total of 731 articles were identified, of which, 179 duplicate articles were removed by endnote, and 331 studies were excluded by assessing their title and abstract. Finally, a total of 28 met the inclusion criteria, which were from 17 African countries (Fig 1). Furthermore, from the total of 28 eligible studies, 11 studies [21–31] used MNA tool, whereas the remaining 17 studies [13, 32–47] used BMI to assess undernutrtion in aged population (Table 1).

**Table 1:** Summary of the characteristics of included studies in the review.

S.No	Authors	Year of Publication	Country	Measurements	Study setting	Country’s economy level	Sample size	Prevalence of malnutrition	Quality score
1	Pierre J et.al [35]	2017	Central Africa	BMI	Community-based	Low-income economy	990	19.2	7
2	MB Andre et.al [46]	2013	Republic of Congo	BMI	Community-based	Low-income economy	370	34.3	8
3	Aganiba BA et.al, [39]	2015	Ghana	BMI	Community -based	Lower-middle income	400	18	7
4	Geofrey M. et.al [36]	2021	Zambia	BMI	Community-based	Lower-middle income	135	30.4	7
5	DM Chilima and SJ Ismail [37]	1998	Malawi	BMI	Community-based	Low-income economy	296	30.06	8
6	Joyce K. and Fred B [42]	2004	Uganda	BMI	Community-based	Low-income economy	100	33.3	6
7	Naidoo et.al [41]	2015	South Africa	BMI	Community- based	Upper-middle income	984	1.8	6
8	W.A.O. Afolabi.et.al [47]	2015	Nigeria	BMI	Community-based	Lower-middle income	140	2.9	9
9	Dawit T et.al [33]	2014	Ethiopia	BMI	Community-based	Low-income economy	757	21.9	7
10	Kidest W et.al [43]	2019	Ethiopia	BMI	Community-based	Low-income economy	554	17.1	9
11	Gustave. M et.al [34]	2021	Cameroon	BMI	Community- based	Lower-middle income	599	19.7	7
12	Faith K et.al [45]	2010	Kenya	BMI	Community-based	Low-income economy	152	39.47	8
13	Legesse M. et.al [13]	2019	Ethiopia	BMI	Community-based	Low-income economy	892	17.1	7
14	Agbozo et.al [38]	2018	Ghana	BMI	Community-based	Lower-middle income	120	10	7
15	N. Menadi et.al [44]	2013	Algeria	BMI	Facility-based	Lower-middle income	314	14.01	8
16	Abdu O et.al [25]	2020	Ethiopia	MNA	Community-based	Low-income economy	592	15.5	9
17	Abate T et.al [30]	2020	Ethiopia	MNA	Community- based	Low-income economy	662	26.6	9
18	A. Talhaoui et.al [21]	2019	Morocco	MNA	Facility-based	Lower-middle income	273	5.2	7
19	A.Y. Abdelwahed et.al [27]	2018	Egypt	MNA	Community-based	Lower-middle income	100	16	7
20	Cheserek MJ et.al [40]	2012	East Africa	BMI	Community-based	Low-income economy	573	26.4	7
21	Andia A et.al [23]	2019	Niger	MNA	Community based	Low-income economy	384	7.8	9
22	Z.K. Adhana et.al [31]	2019	Ethiopia	MNA	Community-based	Low-income economy	423	22.7	6
23	Adebusoye LA, et.al [26]	2011	Nigeria	MNA	Facility-based	Lower-middle income	500	7.8	7
24	Hamza SA. et.al [29]	2018	Egypt	MNA	Community based	Low-income economy	170	26.5	7
25	S.A.Fattah Badr et.al [28]	2019	Libya	MNA	Facility-based	Upper-middle income	312	11.5	9
26	E.M. Mahfouz et.al [22]	2013	Egypt	MNA	Community- based	Lower-middle income	350	8.6	8
27	Nzeagwu O.C &Ozougwu, C.B [32]	2019	Nigeria	BMI	Community based	Lower-middle income	238	1.7	8
28	Adebusoye LA et.al [24]	2018	Nigeria	MNA	Facility-based	Lower-middle income	624	2.24	6

### Prevalence of malnutrition in Africa among aged population

**Fig 2:**
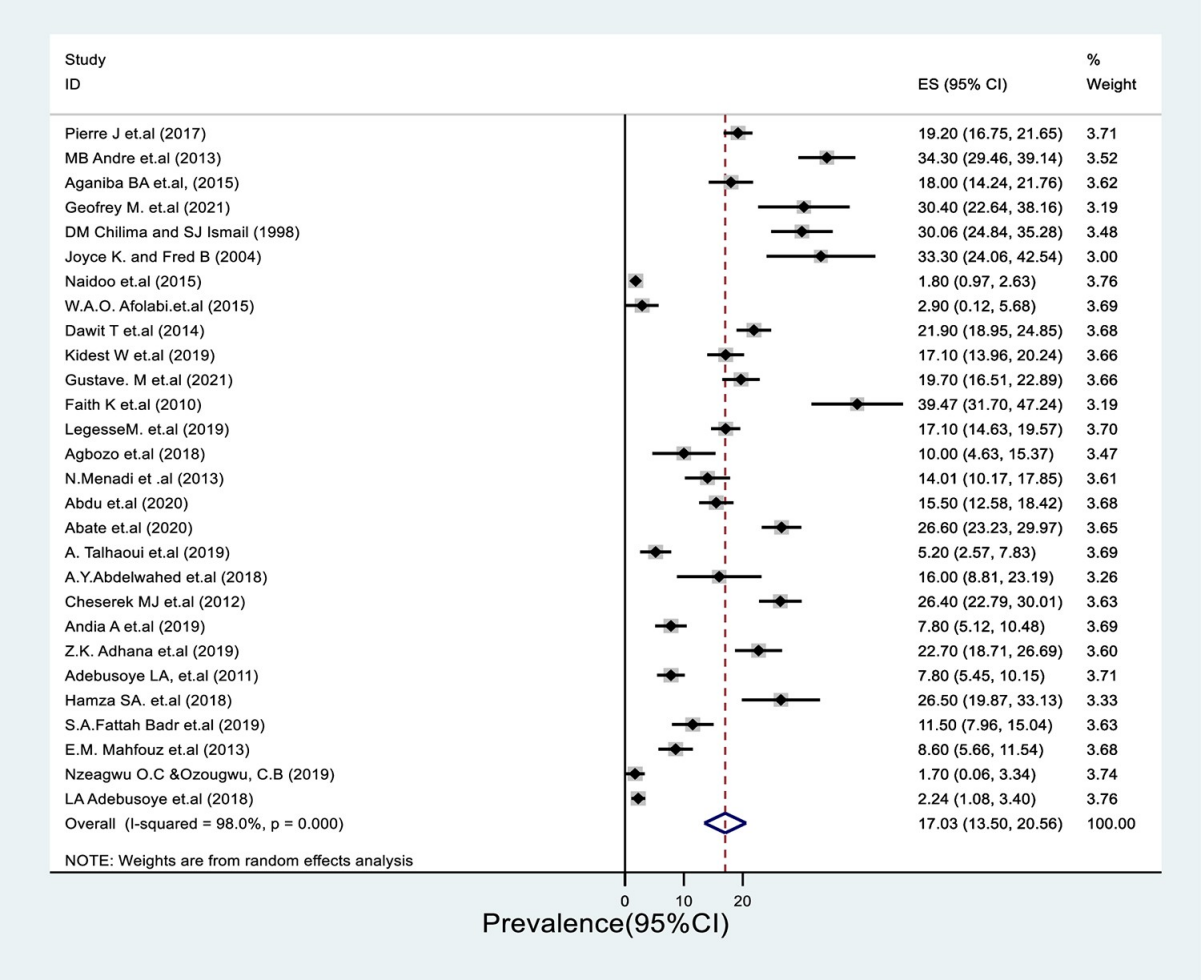
Forest plot of all included studies to assess the pooled prevalence of malnutrition among aged population in Africa.

The pooled prevalence of under-nutrition in Africa is 17% (95%CI; 13.5–20.6) with a significant statistical heterogeneity (I^2^ = 98, P = 0.00) (Fig 2). Substantial variation of prevalence of under nutrition among advanced age population was observed among countries ranging from 1.8% (95%CI; 0.96–2.63) in South Africa to 39.47% (95%CI; 31.70–47.24) in Kenya (S1 Fig).

**Fig 3:**
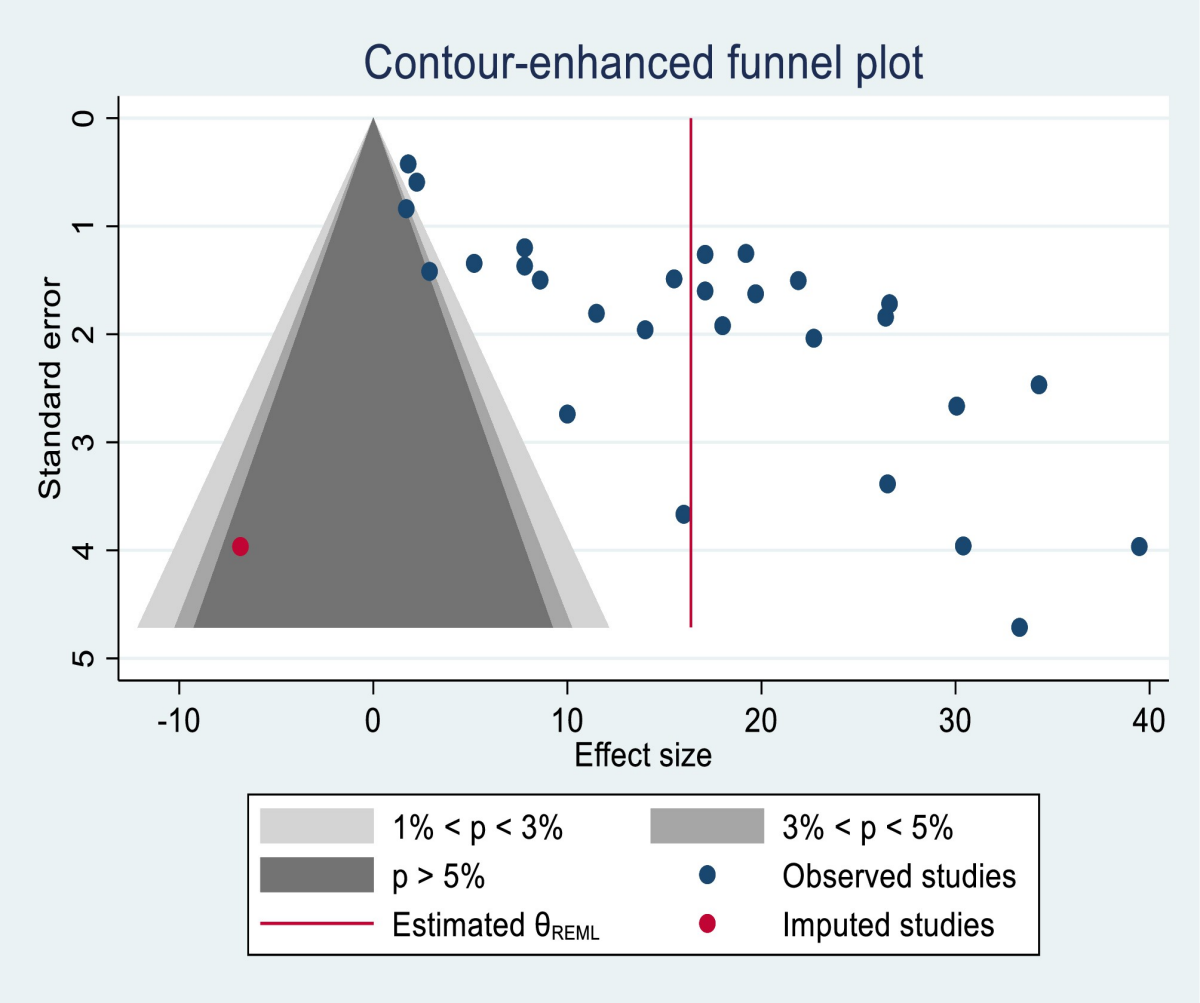
Funnel plot, and trim and fill result of the analysis of included studies.

The sensitivity analysis showed that there were no studies that affect the pooled estimate of malnutrition (S2 Fig). Visual assessment of publication bias was performed using the funnel plot, which revealed asymmetric distribution among studies. In addition, Begg’s test (Pr > |z| = 0.010) and Eggers’ test (P = 0.00) were also carried out that showed the presence of publication bias. Then meta-trim and fill analysis was performed, which quantified the effect of missed studies (Fig 3).

### Subgroup analysis

Subgroup analysis was employed by stratifying using the measurements, study setting, and income levels of the country. The prevalence of malnutrition in aged population was higher in community-based study which was 19.13% (95%CI;- 14.68, 23.59). In addition, it was found to be 19.49% (95%CI;-14.14, 24.84) by using body mass index and 23.56% (95%CI; - 19.68, 27.44) among countries with low-income economies (Table 2).

**Table 2:** Subgroup analysis of pooled prevalence of malnutrition among aged population in Africa.

Variables	Categories	No. of studies	Prevalence (%)	95%CI	I-squared (%)
Measurement	BMI	17	19.49	14.14, 24.84	98.4
	MNA	11	13.42	13.3, 18.27	97
Study setting	Community-based	23	19.13	14.68, 23.59	98.2
	Facility-based	5	7.93	3.73, 12.13	93.4
Income level	Low-income economies (LIE)	14	23.56	19.68, 27.44	93.9
	Lower-middle-income economies (LMIE)	12	10.78	7.06, 14.50	95.6
	Upper-middle-income economies (UMIE)	2	6.49	-3.01,15.99	96.3
Pooled prevalence		28	17.03	13.50, 20.56	98

### Meta-regression

**Fig 4:**
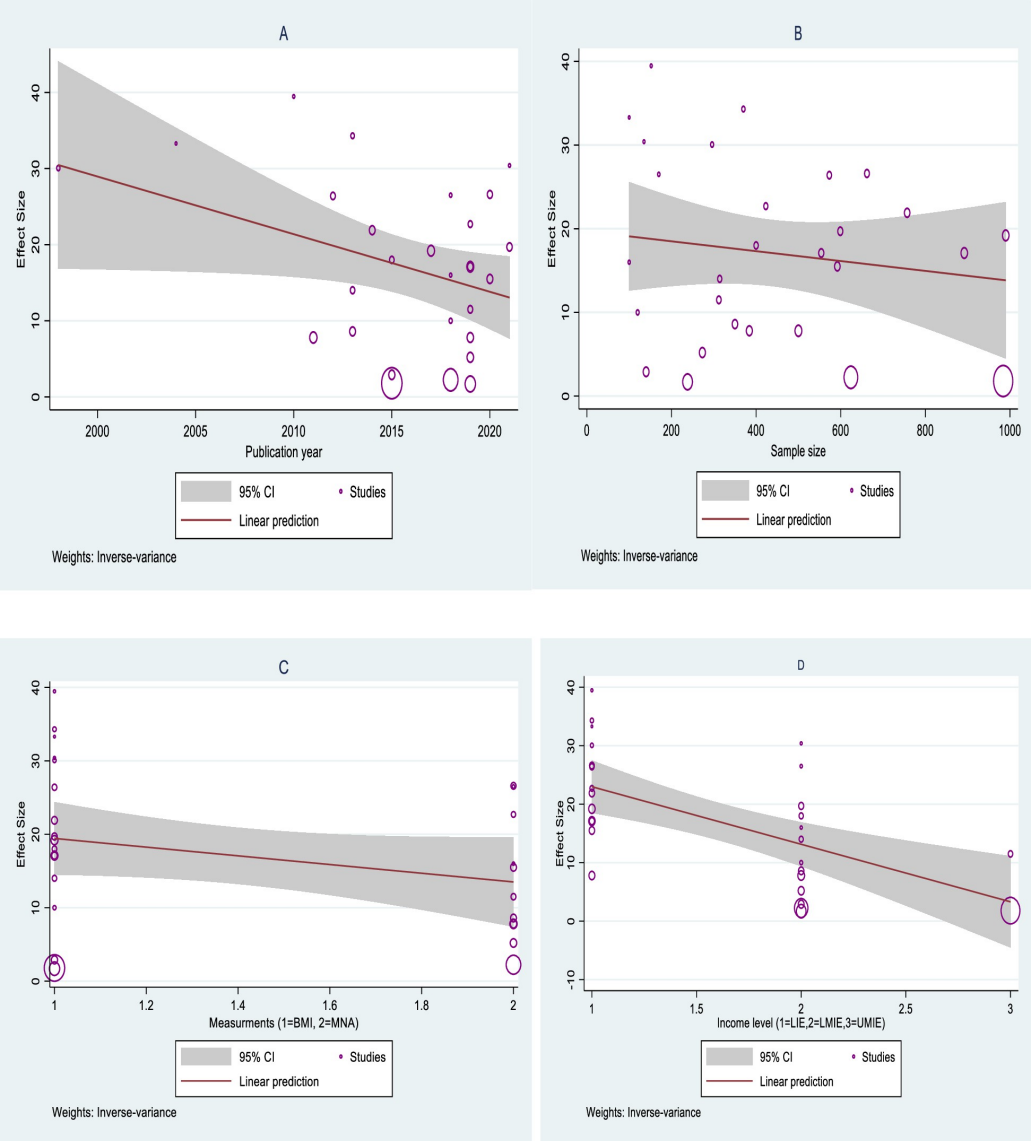
Linear relationship between malnutrition and selected predictors.

A meta-regression was also conducted to identify the relationship between the prevalence of under-nutrition among the advanced age population in Africa and different predictors such as study period, sample size, measurement, and countries’ income level. The meta-regression revealed that the publication year was statically significant, meaning that the prevalence of under-nutrition was steadily decreasing through time (P = 0.04). According to the World Bank’s classification of African countries’ income levels [17], the prevalence of malnutrition among the geriatric population was higher in low-income economies than in lower-middle and upper-middle income economies (P>|z| = 0.00). In addition, malnutrition in Africa among aged population was significantly decreased over the last twenty years (P>|z| = 0.04). However, the relationship between malnutrition with sample size, and measurement was not statistically significant (Table 3, Fig 4).

**Table 3:** Univariate meta-regression analysis result for prevalence of malnutrition among aged population in Africa.

S.No	Variables	P>|z|	*ß*-Coefficient (95%, CI)
1	Study period	0.04	-0.75 (-1.49, -0.01)
2	Sample size	0.44	-0.01 (-0.02, 0.01)
3	Measurements	0.13	-5.92 (-13.76, 1.91)
4	Income level	0.00	-9.84 (-14.97, -4.70)

## Discussion

In this systematic review and meta-regression, we found that malnutrition is a public health problem among aged population in Africa, and the pooled prevalence is estimated to be 17.03%. This finding is higher than the studies conducted in China (3.2%) [48], India (9.1%) [49], developed countries (3.1%) [50]. This discrepancy could be because elders who are living in Africa are in a state of food insecurity and are neglected by the healthcare system as well as limited facilities for elders. However, it was lower than the study conducted on free-living elders in Europe (53%) [51]. This probably due to the fact that those advanced age group might get family support and lower prevalence of non-communicable diseases than free-living people in Europe. This review showed that one in five geriatric people had a low body mass index (<18.5mg/m^2^), and three out of twenty geriatric people had a low MNA (<17 score) living in Africa. The prevalence of malnutrition varies from country to country, which ranges from 1.8 percent in South Africa to 39.47 percent in Kenya. This could be due to socio-economic and healthcare system differences between South Africa and Kenya.

In addition, the prevalence of malnutrition among advanced age group population living in Africa is higher in low income countries as compared with upper-middle income countries. This finding is supported by the study conducted in developed countries [52]. This might be because countries with low economic status could influence people’s food choices, increase the burden of food insecurity, and deteriorate nutritional status of people in those countries. Along with this, those countries with low economic status could not have enough capital to prevent malnutrition among their aged population. Concerning the study setting, the prevalence of malnutrition was higher among community based studies than facility based studies. This could be the encouragement of aged population care and better health services in facilities.

Although substantial improvement in the prevalence of malnutrition has been observed, this problem is still a public health issue. These could be a different factors, for instance aged population are risky for lack of ability to chew and swallow foods, and loss of appetites that lead to malnutiriion. As a result, specific strategies to prevent malnutrition among these neglected segment of the population should be a priority to integrate with existing healthcare systems and nutrition programs.

The strength of this review could be the baseline for nutrition program implementers and policymakers to propose possible policies to integrate with the existing health care system based on the prevalence of malnutrition among advanced age group. Whereas, the limitations were determinants of malnutrition are not addressed, and articles published other than English language were not included in this review. In addition, we used of both (MNA and BMI) measurements that might be affect the estimation of prevalence of malnutrition. Future researchers should include the possible determinants. Hence, the governments of African countries should give attention to the advanced age population, which will be doubled in the near future because of the increments in life expectancy in developing countries.

Nowadays, policymakers, program implementers, and nongovernmental organizations are focusing on maternal and child nutrition. However, this review showed that there is a high prevalence of malnutrition among the aged population. As a result, this underserved population should be targeted and/or integrated into maternal and child nutrition programs, and facilities should be established for aged population care services. National and international policy arena, nutritional intervention focusing on prevention and treatment of malnutrition among the aged population should be considered.

## Supporting information

S1 Fig Pooled prevalence of malnutrition among African countries. (TIF)

S2 Fig Sensitivity analysis plot for the pooled prevalence of malnutrition among aged population in Africa. (TIF)

S1 Table PRISMA 2020 checklist. (DOCX)

## References

[1] Biritwum RB, Yawson A. E., Mensah G., & Minicuci N. Ghana: study on global ageing and adult health (SAGE) wave 1 national report. ResearchGate. Epub ahead of print. 2015.

[2] WHO. Global Health and Aging. World Health Organisation, Geneva, Switzerland. 2011.

[3] de Morais C OB, Afonso C, Lumbers M, Raats M, de Almeida MDV. Nutritional risk of European elderly. Eur J Clin Nutr 2013;67:1215–9. doi: 10.1038/ejcn.2013.175 24065060

[4] van Bokhorst-de van der Schueren MA L-MS, de Vries OJ, Danner SA, Kramer MH, Muller M. Prevalence and determinants for malnutrition in geriatric outpatients. Clin Nutr 2013;32:1007–11. doi: 10.1016/j.clnu.2013.05.007 23755842

[5] Fávaro-Moreira NC K-HS, Matthys C, Vereecken C, Vanhauwaert E, Declercq A, Bekkering GE, et al. Risk Factors for Malnutrition in Older Adults: A Systematic Review of the Literature Based on Longitudinal Data. Adv Nutr. 2016 May 16;7(3):507–22. doi: 10.3945/an.115.011254 27184278 PMC4863272

[6] Deer RR HE, Mera A, Howe K, Goodlett S, Robertson N, Volpi E. Dietary intake patterns of community-dwelling older adults after acute hospitalization. J GerontolSeries A. 2022;77(1):140–7. doi: 10.1093/gerona/glab232 34410002 PMC8923293

[7] Hernández-Galiot A GI. Quality of life and risk of malnutrition in a home-dwelling population over 75 years old. Nutrition. 2017;35:81–6. 28241994 10.1016/j.nut.2016.10.013

[8] S. J. The importance of nutrition and preventing malnutrition in older adults: a literature review and informational booklet. Undergraduate Honors Thesis. 2021; University of Nebraska-Lincoln.

[9] Ngatia EM GL, Macigo FG, et al. Nutritional status and oral health of elderly population in Nairobi. East Afr Med J. 2008;85(8):378–385. 19115555 10.4314/eamj.v85i8.9655

[10] Ferdous T KZ, Wahlin Å, Streatfield K, Cederholm T. The multidimensional background of malnutrition among rural older individuals in Bangladesh–a challenge for the millennium development goal. Public Health Nutr. 2009;12:2270–8. doi: 10.1017/S1368980009005096 19257922

[11] Baweja S AH, Mathur A, Haldiya KR and Mathur. Assessment of nutritional status and related risk factors in community dwelling elderly in western Rajasthan. Journal of Indian Academy of Geriatrics 2008; 1: 5–13.

[12] WHO, Global health and Aging, D.o.H.a.H. Services, Editor 2016, National Institute on Aging/Health, USA: USA.

[13] Legesse M, Abebe Z, Woldie H. (2019) Chronic energy deficiency and associated factors among older population in Ethiopia: Acommunity based study.PLoSONE14(4):e0214861. doi: 10.1371/journal.pone.0214861 PMC6457535 30969978

[14] Page MJ, McKenzie JE, Bossuyt PM, Boutron I, Hoffmann TC, Mulrow CD, et al. The PRISMA 2020 statement: an updated guideline for reporting systematic reviews. BMJ 2021;372:n71. doi: 10.1136/bmj.n71 33782057 PMC8005924

[15] Flegal KM, Kit BK, Graubard BI. Body mass index categories in observational studies of weight and risk of death. Am J Epidemiol. 2014;180(3):288–96. doi: 10.1093/aje/kwu111 24893710 PMC4732880

[16] Nestle. (2009). Mini Nutritional Assessment Form. [Online] Available: www.mnaelderly.com/practice/forms/MNA_english.pdf.

[17] Nada Hamadeh, Catherine Van Rompaey, Eric Metreau; New World Bank country classifications by income level: 2021–2022.

[18] Higgins JP TS, Deeks JJ, Altman DG. Measuring inconsistency in meta-analyses. Bmj. 2003;327(7414):557–60. doi: 10.1136/bmj.327.7414.557 12958120 PMC192859

[19] Huedo-Medina TB, Sanchez-Meca J, Marın-Martınez F, Botella J. Assessing heterogeneity in meta analysis: Q statisticor I2 index? Psychological methods. 2006;11(2):193. doi: 10.1037/1082-989X.11.2.193 . 16784338

[20] Duval S, Tweedie R. A nonparametric “trim and fill” method of accounting for publication bias in meta-analysis. Journal of the american statistical association. 2000;95(449):89–98.

[21] Talhaoui A., Aboussaleh Y., Ahami A., Sbaibi R. and Agoutim N. (2019) Association between Malnutrition and Cognitive Impairment among Morocco’s Older Adults. Open Journal of Medical Psychology, 8, 1–14. 10.4236/ojmp.2019.81001.

[22] Mahfouz E.M., Mohammed E.S., Abd El-Rhman T.A. Assessment of nutritional statutes of elderly population in rural Minia, Egypt. Journal of Aging Research & Clinical Practice. Volume 2, Number 3, 2013.

[23] Andia A, Fourera S, Souleymane B, Mamane D, Adehossi E. Evaluation of Nutritional Status at Household in Elderly Assessed by Mini Nutritional Assessment (MNA) in West Africa Country, Niamey-Niger. Am J Gerentol Geriatr. 2019; 2(1): 1018.

[24] Adebusoye LA, Ogunbode AM, Olowookere OO, Ajayi SA, Ladipo MM. Factors associated with sarcopenia among older patients attending a geriatric clinic in Nigeria. Niger J Clin Pract 2018;21:443–50. doi: 10.4103/njcp.njcp_374_17 29607855

[25] Abdu Abdu Oumer, Yimamu Imam Dagne, Kahsay AA. Predictors of malnutrition among older adults aged above 65 years in eastern Ethiopia: neglected public health concern. BMC Geriatrics, 10.1186/s12877-020-01911-2 2020. PMC7684913 33228614

[26] Adebusoye LA, Ajayi IO, Dairo MD, AO O. Factors associated with undernutrition and overweight in elderly patients presenting at a primary care clinic in Nigeria.S Afr Fam Pract 2011;53(4):355–360.

[27] Amal Yousef Abdelwahed, Algameel MMM, Tayel DI. Effect of a Nutritional Education Program on Nutritional Status of Elderly in Rural Areas of Damanhur City, Egypt. International Journal of Nursing Science 2018, 8(5): 83–92 doi: 10.5923/j.nursing.20180805.02

[28] Safaa Abd El Fattah Badr, Ali Ateia Elmabsout, Allah IA. Family Support, Malnutrition and Barriers to Optimal Dietary Intake among Elderly Diabetic Patients in Benghazi, Libya. 2019.

[29] Hamza Sara A., Abdul-Rahman Samia A., Nabiel Asmaa M., Sedky AS. Nutritional Status and Health-Related Quality of Life Among Elderly in Rural Area in Egypt. The Egyptian Journal of Geriatrics and Gerontology· October 2018 doi: 10.21608/ejgg.2018.30904

[30] Abate Tadele, Mengistu Berhanu, Atnafu Asmamaw, Derso T. Malnutrition and its determinants among older adults people in Addis Ababa, Ethiopia. BMC Geriatrics. doi: 10.1186/s12877-020-01917-w 33228557 PMC7684921

[31] Adhana Z.K., Tessema G.H., Getie GA. prevalence of under nutrition and its associated factors among old people in Debre Markos town, Northwest Ethiopia 10.14283/jarcp.2019.4.

[32] Nzeagwu O.C and Ozougwu C.B Nutritional and health status of older persons aged ≥ 60 years in rural communities of udi local government area, enugu state, Nigeria. Journal of Dietitians Association of Nigeria (JDAN) Volume 10, November 2019.

[33] Tessfamichael D, Gete AA, Wassie MM (2014) High Prevalence of Undernutrition among Elderly People in Northwest Ethiopia: A Cross Sectional Study. J Nutrition Health Food Sci 2(4): 1–5. 10.15226/jnhfs.2014.00131.

[34] Mabiama G., Adiogo D., Preuxet al P.M., Nutritional status and associated factors among community-dwelling elderly, Clinical Nutrition ESPEN, doi: 10.1016/j.clnesp.2021.08.021 34620321

[35] J esus P, et al., Undernutrition and obesity among elderly people living in two cities of developing countries: Prevalence and associated factors in the EDAC study, Clinical Nutrition ESPEN (2017), 10.1016/j.clnesp.2017.05.007. 30014868

[36] Maila Geofrey, Audain Keiron & Marinda Pamela A(2021) Association between dietary diversity, health and nutritional status of older persons in rural Zambia, South African Journal of Clinical Nutrition, 34:1, 34–39, doi: 10.1080/16070658.2019

[37] Chilima DM and Ismail SJ. Anthropometric characteristics of older people in rural Malawi;European Journal of Clinical Nutrition (1998) 52,643± 649. doi: 10.1038/sj.ejcn.1600617 9756120

[38] Agbozo F, Amardi-Mfoafo J, Dwase H, Ellahi B. Nutrition knowledge, dietary patterns and anthropometric indices of older persons in four peri-urban communities in Ga West municipality, Ghana. Afri Health Sci. 2018;18(3): 743–755. 10.4314/ahs.v18i3.33. PMC6307008 30603008

[39] Aganiba BA, Owusu WB, Steiner-Asiedu M, S. D. Association Between Lifestyle And Health Variables With Nutritional Status Of The Elderly In The Northern Region Of Ghana. 2015.

[40] Cheserek MJ, Tuitoek PJ, Waudo JN, Msuya JM, JK. K. Anthropometric characteristics and nutritional status of older adults in the Lake Victoria Basin of East Africa: region, sex, and age differences,S Afr J Clin Nutr 2012;25(2):67–72.

[41] Naidoo I., Karen E. Charlton, Esterhuizen T, Cassim B. High risk of malnutrition associated with depressive symptoms in older South Africans living in KwaZulu-Natal, South Africa: a cross-sectional survey; Journal of Health, Population and Nutrition (2015) 33:19, doi: 10.1186/s41043-015-0030-0 2015. 26825267 PMC5026002

[42] Kikafunda Joyce K., Lukwago FB. Nutritional status and functional ability of the elderly aged 60 to 90 years in the Mpigi district of central Uganda; Nutrition 21 (2005) 59–66. doi: 10.1016/j.nut.2004.09.009 15661479

[43] Wondiye Kidest, Netsanet Abera Asseffa Tsegaye Demisse Gemebo, Astawesegn FH. Predictors of undernutrition among the elderly in Sodo zuriya district Wolaita zone, Ethiopia.BMC Nutrition(2019) 5:50. doi: 10.1186/s40795-019-0320-9 32153963 PMC7050837

[44] Menadi N, Khaled M. B., Merrakchi, Belbraouet S. Nutritional Status of Elderly People Living at Home in Sidi-Bel-Abbes (West Algeria). Food and Nutrition Sciences, 2013, 4, 860–865 10.4236/fns.2013.48112.

[45] Munoru FK. Dietary And Care Practices, Morbidity And Nutritional Status Of The Elderly In Igembe South, Meru County, Kenya, 2010.

[46] Andre Muzembo Basilua, Dumavibhat Narongpon, Ngatu N’landu Roger, Eitoku Masamitsu, Hirota Ryoji, Suganuma N. Mini Nutritional Assessment and functional capacity in community-dwelling elderly in Rural Luozi, Democratic Republic of Congo; Geriatr Gerontol Int 2013;13:35–42. doi: 10.1111/j.1447-0594.2012.00852.x 22530787

[47] Afolabi W.A.O., Olayiwola I.O., Sanni S.A., Oyawoye O. Nutrient Intake And Nutritional Status Of The Aged In Low Income Areas Of Southwest, Nigeria; Journal of Aging Research & Clinical Practice, Published online February 26, 2015, 10.14283/jarcp.2015.51.

[48] ShiR., DuanJ., DengY., TuQ., CaoY., ZhangM., et al; Nutritional Status Of An Elderly Population In Southwest China: A Cross-Sectional Study Based On Comprehensive Geriatric Assessment, The Journal Of Nutrition, Health & Aging.10.1007/s12603-014-0471-y25560813

[49] Konda Satyanarayana, Ravi Kumar B. P., Purushottam A Giri; Prevalence of malnutrition and its determinants in an elderly people in South India; Vol 5, No 8 (2018), 10.18203/2394-6040.ijcmph20183100.

[50] Cereda E, Pedrolli C, Klersy C, Bonardi C, Quarleri L, Cappello S, et al. Nutritional status in older persons according to healthcare setting: A systematic review and meta-analysis of prevalence data using MNA®. Clin Nutr. 2016 Dec;35(6):1282–1290. doi: 10.1016/j.clnu.2016.03.008 Epub 2016 Apr 6. 27086194

[51] de Morais C, Oliveira B, Afonso C, Lumbers M, Raats M, de Almeida MD. Nutritional risk of European elderly. Eur J Clin Nutr. 2013 Nov;67(11):1215–9. doi: 10.1038/ejcn.2013.175 Epub 2013 Sep 25. 24065060

[52] Cereda E, Pedrolli C, Klersy C, Bonardi C, Quarleri L, Cappello S, et al. Nutritional status in older persons according to healthcare setting: A systematic review and meta-analysis of prevalence data using MNA®. Clin Nutr. 2016 Dec;35(6):1282–1290. doi: 10.1016/j.clnu.2016.03.008 Epub 2016 Apr 6. 27086194

