# Supplementary figures and images for: Malnutrition among the aged population in Africa: A systematic review, meta-analysis, and meta-regression of studies over the past 20 years
Source: PLoS One. 2022 Dec 9;17(12):e0278904. doi: 10.1371/journal.pone.0278904 (PMC9733888; doi:10.1371/journal.pone.0278904)

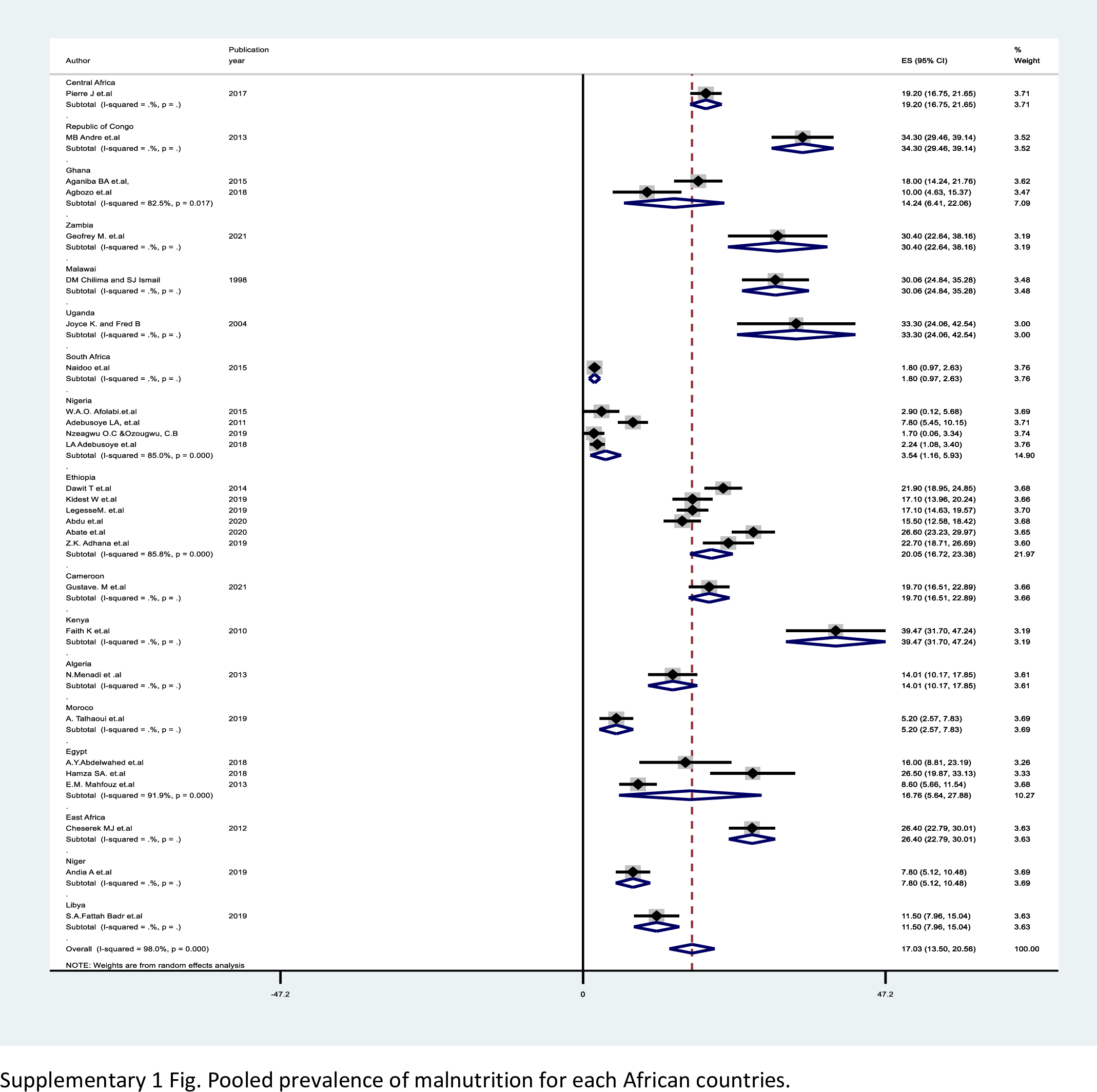

Supplement: S1 Fig — (TIF) [file pone.0278904.s001.tif]

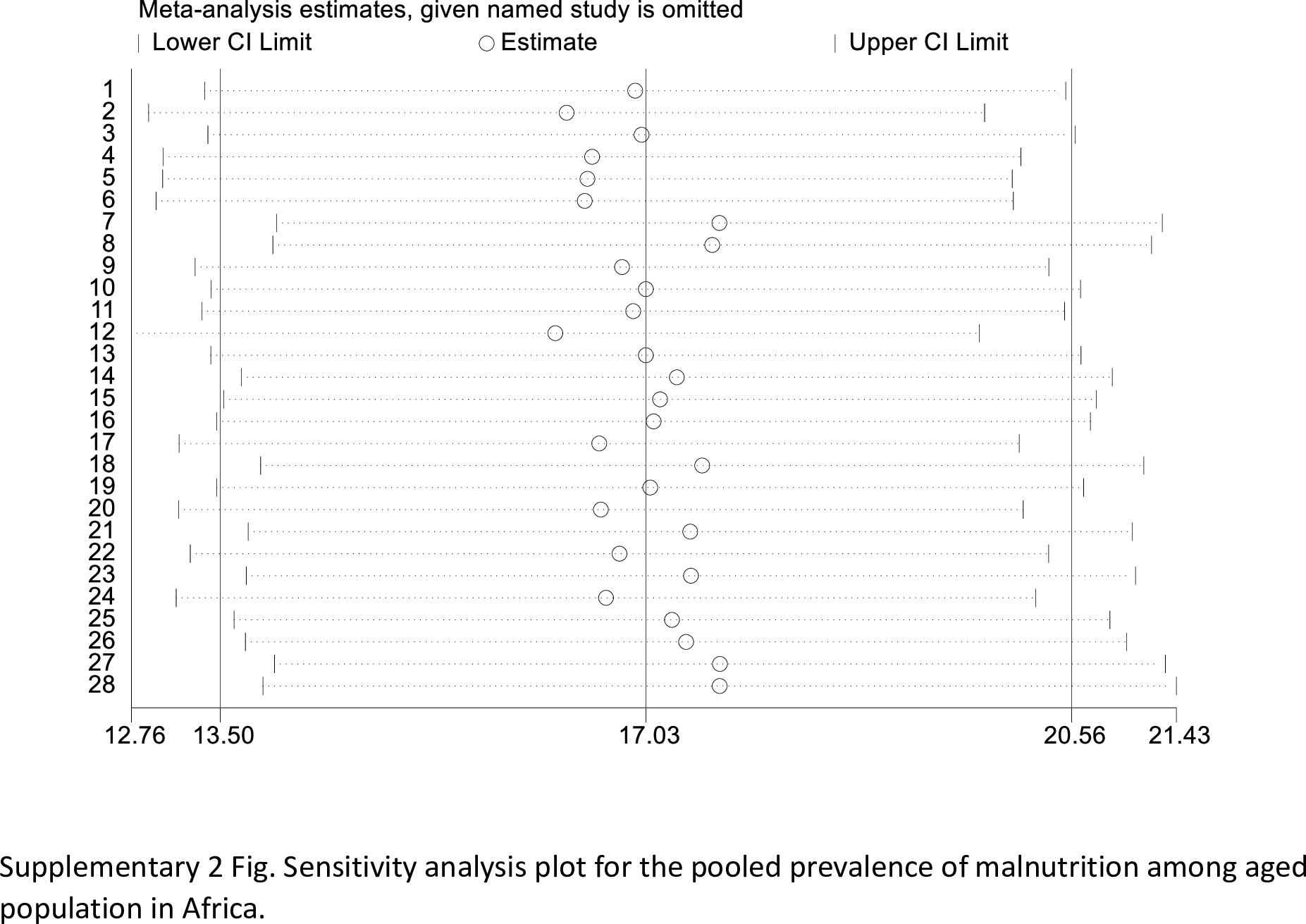

Supplement: S2 Fig — (TIF) [file pone.0278904.s002.tif]
