# Supplementary material for: Malnutrition among the aged population in Africa: A systematic review, meta-analysis, and meta-regression of studies over the past 20 years
Source: PLoS One. 2022 Dec 9;17(12):e0278904. doi: 10.1371/journal.pone.0278904 (PMC9733888; doi:10.1371/journal.pone.0278904)
Supplement: Attachment — Submitted filename: Response_To_Reviewers.docx [file pone.0278904.s004.docx]

| S/No | Reviewers comment | Authors response | Reference |
| --- | --- | --- | --- |
|  | Reviewer #1 |  |  |
| 1 | Good effort. Well-compiled and systematically presented. Commendable job. The article is Technically sound. The objectives are attained. The data and information needed are all available. The language is simple and lucid. | We are grateful for your suggestion. | ----- |
|  | Reviewer #2 |  |  |
| 1 | #51 Key terms: Please add meta-analysis | Thank you very much for your comments. As per the comment it has been corrected. | Page 3 |
| 2 | #81 what happens to the other publications not in English? Will this not result in publication bias? Some eligible publications may be in other languages other than English | We appreciated the reviewer’s insightful comment and mentioned as one of the limitations of the review. | Page 13 |
| 4 | #213: The limitation should include inability to access articles/studies not written in  English | Thanks for your insightful comments. It has been corrected. | Page 13 |
|  | Reviewer #3 |  |  |
| 1 | Lines 55, 77-79, 84-85, 115, 132, 140-141, 155-157 and 157-158 all are not grammatically correct and need to be corrected | Thank you so much for the comments and it have been corrected one by one. | Page 3, 4,5, 6,7,8&9 |
| 2 | Lines 176-177: The statement is not complete and seems to be hanging | As per the comment it has be corrected | Page 11 |
| 3 | Lines 198-200: The statement need to be rephrased | Thanks! We authors made a correction as per the given comment. | Page 12 |
| 4 | Some of the references needs to be reviewed especially where complete citations were made. There is no need to include DOI number and website where the reference was retrieved after giving full citation already. | We are grateful for the comments and made a correction. But, the submission guideline recommend DOI | Page 15-17 |
| 5 | Figure 1: Showing the PRISMA flow diagram indicated that 28 studies were included in the review, but going through the diagram I noticed a total of 27 studies (25 reports assessed for eligibility from databases and 2 reports assessed for eligibility from other methods). Therefore, this need to be crosschecked and corrected before final submission. | Thanks! It has been crosschecked and corrected. | Figure 1 |
|  | Academic editor comment | Response | Reference |
| 1 | Further clarification should be included why they pooled estimate of under nutrition among older population is a necessity from the two measurements (BMI and MNA). Could it provide precise and valid evidence for determining malnutrition burden? | Thank you very much for such insightful comments. Although MNA measurement is preferable for those segment of the population, both BMI and MNA are recommended to estimate the prevalence of undernutrition. The precision may be affected because of using two measurements together, but they can predict the current undernutrition status among the participants. | Page 5 |
| 2 | The authors should consider two scenario: 1) using a valid measurement tool(single tool) to define their primary outcome of interest, generate the  findings using such indicator (e.g. MNA <17); 2) use additional measurement modalities utilized for malnutrition screening or diagnosis and do sensitivity and subgroup analysis to see the possible heterogeneity. BMI and MNA are highly correlated since BMI is used as one component to compute MNA. | We are grateful for the comments. And based on the comments provided we performed sensitivity and subgroup analysis. Yes, BMI and MNA are highly correlated each other but the result of MNA was selected when the two measurements were used in a single study. | Page 9, 10 |
| 3 | Does the current estimate reflect the prevalence of undernutrition among older age individual? The setting of the study must be specified to link the current estimate with what is supposed to be (facility based or community based). | Thanks! Yes the current estimate can estimate the prevalence of undernutrition, but it might be affected with some limitations like not included those studies which published other than English language. We added a subgroup analysis (facility based or community based). based on the given comments | Page 10 |
| 4 | The discussion section is not analytically organized, shallow and should forward the practical implication of the study and limitation needs some additional  amendment. | We are grateful for the comments. It has been corrected. | Page 11-13 |
| 5 | The author should mention the assumptions used in the meta-regression. | Thanks! We put the assumptions in the analysis section. | Page 6 |

**Authors’ response**

We are grateful for the comments and suggestions raised by the reviewers and editor, and the response for each comment are explained and corrected in the main document.
